# Supplementary material for: Complexome profiling of the Chlamydomonas psb28 mutant reveals TEF5 as an early PSII assembly factor
Source: Plant Cell. 2025 Mar 17;37(6):koaf055. doi: 10.1093/plcell/koaf055 (PMC12164590; doi:10.1093/plcell/koaf055)
Supplement: koaf055_Supplementary_Data [file koaf055_supplementary_data.zip › psb28_interactive.zip!/plots/intensity/qualityPlots/peptides/PDK3_Cre05.g241750.t1.2.html]

### Cre05.g241750.t1.2

Trivial name: PDK3  
SeqCoverage [median]: 15.20  
Mass: 44.59 kDa  
  
 

| Sequence | MissedCleavage | Mass [Da] | wtIntensity | muIntensity | Score |
| --- | --- | --- | --- | --- | --- |
| FADSDDAAPPIR | 0 | 1273.59 | 0.00 | 307.83 | 75.29 |
| LVVAEGGEDVTLK | 0 | 1328.72 | 436.30 | 934.80 | 75.23 |
| VSDEGGGIPR | 0 | 985.48 | 680.12 | 823.69 | 88.95 |
|  |  |  | 1116.4 | 2066.3 |  |
